# The Driving Mechanisms of Soil Microbial Community Diversity and Stability in Different Plant Communities Along the Lower Jinsha River’s Water-Level-Fluctuation Zone

**DOI:** 10.3390/microorganisms14030604

**Published:** 2026-03-09

**Authors:** Jingying Lu, Yuehua Zhang, Xianyong Dong, Xiaogang Wu, Lumei Xiao, Kaiwen Pan, Lin Zhang, Juan Wang

**Affiliations:** 1 China Three Gorges Construction Engineering Corporation, Beijing 101100, China; lu_jingying@ctg.com.cn (J.L.); dong_xianyong@ctg.com.cn (X.D.); 2 Chengdu Ecological Environment Monitoring Center Station of Sichuan Province, Chengdu 610000, China; zhangyuehua21@mails.ucas.ac.cn (Y.Z.); 3 Ecological Restoration and Biodiversity Conservation Key Laboratory of Sichuan Province, Chengdu Institute of Biology, Chinese Academy of Sciences, Chengdu 610213, China; wuxg@cib.ac.cn (X.W.); xiaolm@cib.ac.cn (L.X.); pankw@cib.ac.cn (K.P.)

**Keywords:** water-level-fluctuation zone, soil nutrient, microbial communities diversity and stability, co-occurrence network patterns

## Abstract

The Water-Level-Fluctuation Zones (WLFZ) of the Lower Jinsha River, as a typical transition areas between land and water, show crucial ecological functions. However, the relationship between soil nutrients and microbial communities in different plant communities of the WLFZ is poorly understand. Therefore, we chose four typical plant communities, including *Parthenium hysterophorus* (*P. hysterophorus*), *Ziziphus mauritiana* (*Z. mauritiana*), *Cynodon dactylon* (*C. dactylon*), *Zea mays* (*Z. mays*), as a long-term plant communities experiment-monitoring site in a WLFZ of the Lower Jinsha River. By using high-throughput sequences, Mantel test and Mediation model, we explored the changing characteristics of soil nutrients and microbial communities, especially bacteria and fungi, and their driving role in the microbial stability in four typical plant communities. The results indicated that soil properties and enzyme activities noticeably changed among four types of different plant communities in the WLFZ, of which their *P. hysterophorus* and *Z. mauritiana* treatments were eventually higher than their of *Z. mays* and *C. dactylon* treatments. In the bacteria and fungi communities, the OTU number of *P. hysterophorus* and *Z. mauritiana* treatments were higher than their of *C. dactylon* and *Z. mays* treatments, which showed that the bacterial biomarkers only explained with the order, but the fungal biomarkers could explain with species. The bacterial and fungal diversity among four types of different plant communities in the WLFZ significantly changed such that the bacterial and fungal explanations of principal coordinate analysis (PCoA) was at 42.45% and 28.17%, respectively, and the anosim analysis of bacteria and fungi showed the *p* was 0.001 and the R was at 0.6995 and 0.7491. The bacterial and fungal co-occurrence network patterns presented that the bacterial community structure of the *C. dactylon* and *P. hysterophorus* treatments were the most complicated under the *Z. mauritiana* and *Z. mays* treatments, whereas the communities stability of *C. dactylon* and *P. hysterophorus* treatments were notably lower than that of their *Z. mauritiana* and *Z. mays* treatments. Lastly, the CCA, mantel test and intermediary analysis indicated pH served as the primary direct driver in the *Z. mauritiana* community, soil moisture exerted dominant effects in *Z. mays* and *P. hysterophorus*, while in *C. dactylon*, bacterial stability was indirectly modulated by pH mediated through SMC changes. This study highlights the major role of soil nutrients and enzyme activities in driving ecosystem stability of bacterial and fungal communities in four different plant communities in the WLFZ.

## 1. Introduction

The Water-Level-Fluctuation Zone (WLFZ) is a critical ecosystem that bridges terrestrial and aquatic environments [1,2], characterized by the periodic submersion and exposure due to natural and anthropogenic factors [3,4]. This dynamic zone plays a pivotal role in energy exchange and acts as a natural barrier against pollution entering water bodies [5,6]. However, the unique conditions of the WLFZ, marked by periodic submersion and exposure, create both challenges and opportunities for ecological management [7,8]. One significant challenge is the formation of large areas of vacant habitat within the WLFZ, which can be rapidly colonized by plants with strong dispersal abilities and high colonization rates [6,9]. On the other hand, the WLFZ presents a valuable opportunity for human intervention [10]. These plants, whether introduced through human intervention or natural processes, can significantly alter the local ecosystem [11,12]. Prior research indicated that the growth of vegetation and well-planned agricultural practices can lead to the squeezing out of existing habitats, potentially reducing biodiversity [9,13]. Moreover, the presence of these plants can influence the transformation and release of soil nutrients, which in turn affects the soil’s microbial communities, thereby exacerbating the ecological vulnerability of the WLFZ [13,14,15]. For the moment, a large number of researchers have focused on the soil nutrients and enzyme activities of periodic flooding in the WLFZ [2,4], and there are few studies on the soil nutrients, enzyme activities, microbial communities’ characteristics and their significant associations with different plant communities in the WLFZ.

The soil nutrient status of the WLFZ is regulated not only by the periodic submersion and exposure, but also by the effect of different plant communities [16,17]. Meanwhile, different plant communities could cause a succession change in microbial activity, soil enzyme activities, and changes in the root micro-environment, thereby affecting the soil nutrition of fixation, mine-ralisation, and loss, especially the agricultural vegetation of cultivating significantly effect the soil nutrition status of the WLFZ [13,15]. In the case of the Yangtze River riparian zone, relative to bare soil, vegetation covers enhanced the soil organic carbon (SOC) and total nitrogen (TN), and the supply and retention of soil-effective nutrients under different vegetation were improved, and different microbial communities were shaped [18,19]. At the same time, the research of the Three Gorges Reservoir indicated that the SOC, TN and AN concentrations were notably higher in the topsoil than in the deep soil [1,20]. Soil enzymes, as catalysts for soil nutrient activation, turnover and supply, are involved in a variety of ecological process and material cycling in the soil, and can be an important indicator of soil nutrient supply capacity [13,21,22]. The research of aquatic–terrestrial ecotones of Caohai Lake in southwest China showed that the soil enzyme activities of the dry–wet cycling zone increased significantly to compare with the severe inundation zone [16,23]. Though the soil nutrient changes in the WLFZ had many reports, only a few studies referred to soil enzyme activity [7,24], which to a certain extent limited deeply understanding of the mechanism of soil nutrient transformation and transport in the WLFZ of Jinsha River.

Soil microorganisms are mainly involved in regulating soil material cycling and biochemical [25], and play a portal role in the soil enzyme, nutrient cycling, and the improvement of soil properties, and also sensitively reflect the heath of soil ecosystems in the WLFZ [11,21]. Prior researchers exploited soil DNA extraction and MiSeq sequencing of 16S rRNA gene amplicons to analyze the structure and composition of prokaryotic communities in the riparian zone of the Three Gorges Reservoir, and discovered that soil properties and water submergence were also an important and critical control factor affecting the ability, structure and composition of prokaryotic communities [1,20]. However, prior research analyzed soil samples of a riparian zone in the Three Gorges Reservoir using DNA extraction, which found that the wetting–drying alternation could change microbial communities, whereas the microbial diversity and stability was no significant difference among the four treatments [26]. As a vital indicator of soil microbial communities characteristics, the functional diversity of soil microbial communities is essential for the display and management of soil ecosystem in the WLFZ [27,28].

A growing number of researchers have investigated the microbial stability under the anthropogenic disturbances and natural conditions [28,29]. Some researchers have shown that soil pH, SOC and TK are closely related with microbial diversities and network topological characteristics [24,30]. A study reported that enhanced soil microbial stability is associated with SOC storage and the network stability of the fungal community is higher than the bacterial community [25]. Moreover, another study found that natural degradation could reduce β diversity of soil microbes and alter composition of soil microbes, thereby significantly reducing soil microbial network complexity and noticeably improving network stability [31]. Although most previous studies had focused on the effects of the external environment on microbial communities’ structure and composition [2,5], the diversity and stability of microbial communities’ structure had not been explored for specific species of vegetation in the WLFZ of the Lower Jinsha River.

Wudongde Reservoir (WR), located on the mainstream of the Jinsha River on the border between Luquan County in Yunnan Province and Huidong County in Sichuan Province, is China’s fourth-largest and the world’s seventh-largest hydropower station [3,7]. Official storage of water began on January 15, 2020. Thereafter, drainage begins in June and storage begins in October each year, and this operation is called summer drainage and winter storage [6,10]. After the reservoir of water level drops, the vegetation (*Parthenium hysterophorus* (*P. hysterophorus*)*,* and *Cynodon dactylon* (*C. dactylon*)) of WLFZ grows back quickly. Meanwhile, residents plant crops (*Zea mays* (*Z. mays*)) in June and harvest by November in the WLFZ. As a result, this can lead to the formation of multiple land uses in the WLFZ [7,10]. In the case of hydroelectric power station water level dispatching, the water level in the reservoir area rises and falls more obviously, which accelerates the process of soil erosion in the ablation zone, and grasps the change rule of soil properties, enzyme activity and microbial community structure in the ablation zone [29,32,33]. This will help to further understand the process of material and energy exchange between soil and water of different plant communities on the floodplain, and at the same time, it can also provide a certain scientific basis for the recovery of vegetation in the floodplain [3].

So far, the ecological study of the WFLZ of the Lower Jinsha River is still in the initial stage, and the existing results mainly focus on the succession of vegetation communities in the floodplain and the screening of restoration plants, and there is a lack of in-depth research on the changes in soil characteristics and microbial communities under different land use modes and vegetation communities [3,7,10]. Therefore, in this study, we took the WFLZ in the reservoir area of Wudongde power station on the Lower Jinsha River as the study area, and compared the naturally restored suitable plants of the WFLZ, such as *P. hysterophorus*, *C. dactylon*, and artificial crops of *Z. mays* and *Ziziphus mauritiana* (*Z. mauritiana*), to explore the effects of the four different communities on the soil nutrients, enzyme activities, and microbial communities composition, so as to get a preliminary grasp of the effects of the four different communities on the soil characteristics and microbiological patterns of this area. Attempts were made to answer the following questions: (1) what are the differences in soil nutrients and enzyme activities among the four different communities; (2) what are the differences in soil microbial composition and diversity among the four different communities; (3) what are the differences in soil microbial community stability and network complexity among the four different communities; and (4) what are the external factors that affect the microbial stability of the four different communities? This study aims to provide a scientific basis for the ecological restoration of vegetation in the reservoir area of the lower Jinsha River.

## 2. Study Area and Material Method

### 2.1. Study Area Overview

The experimental site was located in the entrance portal of Jinsha River and Longchuan River (25.95° N, 101.88° E, elevation 968 m) and situated in Xiaonabie Village, the Huangguayuan Town, Yunnan Province, China. This site represents a typical southern subtropical hot and dry river valley climate, frost-free all year round, with crops twice a year, with an average temperature of 21.5 °C. The area’s average annual precipitation is 656.8 mm, with the rainy season (June–October) precipitation accounting for more than 90% of the annual precipitation, and the annual evaporation is five times the annual precipitation. The soil typology of the experiment site is torrid red soils. Our experiment site was cleaned of crops and had given up farming in 2019 and went through three processes of inundation-drying from 2020 to 2022. The plants *Zea mays*, *Capsicum annuum* and *Ziziphus mauritiana* grew year-round before the WR was used for stored water. Currently, the native plants are mostly heat- and drought-tolerant, such as *Parthenium hysterophorus*, *Cynodon dactylon*, *Heteropogon contortus* and *Dodonaea viscosa*, except for agriculture crops.

### 2.2. Experiment Method

We chose a typical plant community after abandoning farming and crops community of flat topography and similar slopes as a long-term experiment monitoring site in the WFLZ. Then, the long-term experiment monitoring site included four types of plant communities, *C. dactylon* and *P. hysterophorus*, *Z. mays* and *Z. mauritiana*, respectively, and each plant community was spaced 50 m apart and set up in six plots of 1 m × 1 m, with each plot spaced 10 m apart, with the total of plots of 1 m × 1 m being 24 plots. The *Z. mauritiana* community was never submerged by lake water, but was irrigated by local residents, including with urea and ammonium phosphate. Within the typical distribution range of each plant community, six spatially independent plots with sufficient separation (>10 m) were selected based on GIS predictions and field surveys. The *C. dactylon*, *P. hysterophorus*, and *Z. mays* communities were flooded after 2020 without applying any exogenous additives.

**Figure 1:**
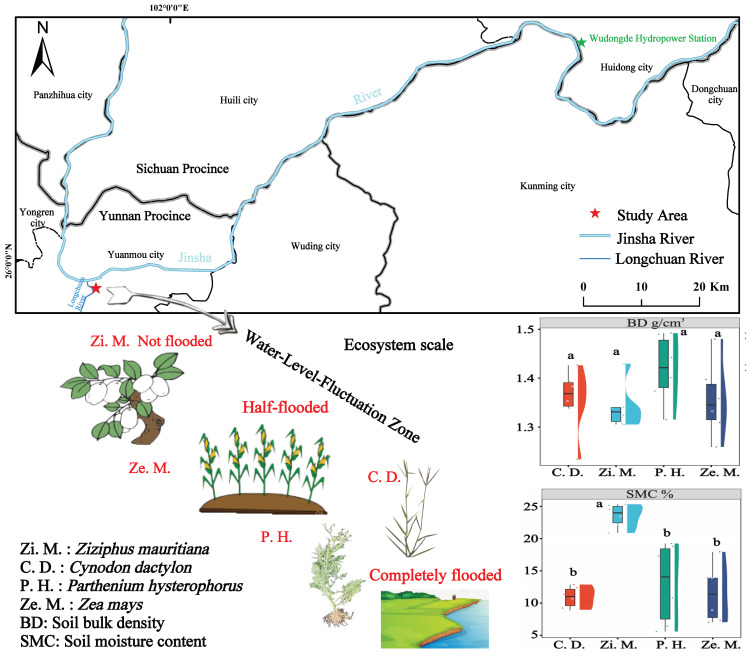
Geographical location of the study area in the WLFA. Notes: C. D.: *Cynodon dactylon*; Zi. M.: *Ziziphus mauritiana*; P. H.: *Parthenium hysterophorus*; Ze. M.: *Zea mays*. The lowercase letters indicate significance at the *p* = 0.05 level.

Thereafter, we removed surface vegetation, litter and obvious root systems in each plot, and used a 5 cm diameter soil auger on the top layer of soil (0–20 cm) according to the “five-point sampling method”. The five auger soil samples were mixed thoroughly and divided into three soil samples to be stored separately for the determination of basic soil properties after natural air-drying, full grinding and sieving; the determination of soil enzyme activities after sieving through 2 mm sieve and low-temperature preservation was set 4 °C; and the determination of soil microbial high-throughput after preservation was at −80 °C. According to Figure 1 and Table 1, we can recognize that this is the basic situation for the four types of plant communities, especially soil bulk density (BD), soil moisture content (SMC), longitude and latitude, slope and community overview. Among them, there was a significant difference in SMC and the *Z. mauritiana* community was much higher than the other communities, which was due to irrigation.

### 2.3. Indicator Measurement and Methodology

In this study, four plant community types, *P. hysterophorus*, *C. dactylon*, *Z. mays* and *Z. mauritiana*, were determined in the soil nutrients, enzyme activities and microbial high-throughput sequencing sections [34,35].

#### 2.3.1. Methods for Analyzing Indicators of Soil Properties

Soil bulk density (BD) was determined using the ring knife method. The formula for calculating the BD is Formula (1).

(1) $$BD=\frac{\left(M1-M0\right)}{V}$$

V represents the volume of the ring knife; the mass of the empty ring knife with the filter paper is weighed in advance and is recorded as M_0_; the mass of the entire ring knife and the soil is weighed and recorded as M_1_.

Soil moisture content (SMC) was determined using the drying method. Many researchers utilized the differential method to calculate the SMC. The details of the formula are as follows (2):

(2) $$\mathrm{SMC}=\frac{(W1-W2)}{(W2-W0)}\times 100\%$$

Empty aluminum box weight and is represented as W_0_; the weight of the box and fresh soil is recorded as W_1_; it is dried to a constant weight and this weight is recorded as W_2_.

The pH was determined using a pH meter (soil–water ratio of 1:2.5); the SOC content was determined using the K_2_Cr_2_O_7_ volumetric method with external heating; the TN content was determined using the semi-micro-KjeLEfSehl-method; total phosphorus (TP) content was determined by NaOH fusion–molybdenum antimony colourimetric method; total potassium (TK) was determined by sodium hydroxide fusion-flame photometric method; available dissolved nitrogen (AN) content was determined by alkaline dissolution-diffusion method; available phosphorus content was determined by NaHCO_3_ extraction–molybdenum antimony colourimetric method; available potassium content was determined by CH_3_COONH_4_ extraction–lame photometric method [4,30,35].

Soil enzyme activities, which refer to the ability of soil enzymes to catalyze the transformation of substances, is often expressed in terms of the number of catalytic reaction products or substrates remaining per unit of soil per unit of time. In this experiment, five soil enzyme activities were measured: urease activity was determined by sodium phenol colourimetric method; sucrase activity (INV) was determined by 3,5-dinitrosalicylic acid colourimetric method; nitrate reductase activity (NR) was determined by red azo compound–spectrophotometric method; catalase activity (CAT) was determined by potassium permanganate titrimetric method; and alkaline phosphatase activity (ALP) was determined by disodium benzene phosphate colourimetric method [13,22].

#### 2.3.2. Soil Microbial Index

The experimental workflow consisted of three key steps: ① Genomic DNA was extracted using the PowerSoil™ Soil DNA Extraction Kit (QIAGEN GmbH, Hilden, Germany). We precisely weighed 0.5 g of fresh soil sample (stored at −80 °C) for DNA extraction, following the kit instructions. We assessed DNA integrity via 1% agarose gel electrophoresis and determined DNA concentration and purity using a NanoDrop One spectrophotometer (Beijing Starvo Technology Co., Ltd., Beijing, China). We stored the extracted DNA at −20 °C for subsequent experiments. ② We performed PCR amplification on uniformly diluted DNA. The amplification region and specific amplification primers are shown in Table 2. The PCR amplification process first requires pre-denaturation (94 °C, 5 min), followed by 30 cycles of denaturation (94 °C, 30 s), annealing (52 °C, 30 s), and extension (72 °C, 30 s), concluding with a final extension (72 °C, 10 min). Finally, the amplified products were purified and isolated using 1% agarose gel electrophoresis. ③ PCR amplification products underwent paired-end sequencing on the Illumina MiSeq PE300/NovaSeq PE250 platform (Shanghai Meiji Biotechnology Co., Ltd. Shanghai, China). Quality filtering and redundancy removal were performed using Trimmomatic software (version 0.39) to eliminate low-quality sequences from raw data, followed by FLASH assembly to generate high-quality sequences. UPARSE software (Qiime platform) was employed to cluster the high-quality sequences, grouping tags with similarity exceeding 97% into Operational Taxonomic Units (OTUs). The Qiime software (Qiime platform) package was used to select representative sequences for each OTU and perform database alignment annotation. Both bacterial 16S and fungal ITS sequences were aligned using the Qiime platform [14,15].

LEfSe (Linear Discriminant Analysis Effect Size) identifies and interprets biomarkers in high-dimensional data through a multi-step statistical pipeline. Firstly, the Kruskal–Wallis test screens for features with significant abundance differences among groups. Subsequently, pairwise Wilcoxon tests validate these differences between subgroups. Finally, LDA quantifies the effect size of each confirmed biomarker. For microbial diversity analysis, results are often presented as an LDA histogram combined with a cladogram, illustrating both the effect sizes and the evolutionary relationships of the significant taxa [36,37].

We used the Richness, Shannon–Wiener, Simpson, Margalef, Pielou, and Chao 1 to characterize microbe alpha diversity. Subsequently, it can visualize and quantify the differences between beta diversity in microbial communities by the Similarity Analysis (Anosim) and unconstrained Principal Coordinate Analysis (PCoA) of Bray–Curtis. Lastly, the variance decomposition was used to analyze the interpretation of microbial differences between different land-use methods in the WLFZ and the significance by using the permutation test [31,33,38].

Richness is the most fundamental diversity index that only calculates the number of a sample plot and does not consider relative abundance of species.

(3) $$S_{obs}=N$$

The Shannon–Wiener index considered two aspect of species abundance and evenness that calculate the natural logarithm of the relative abundance of single species and multiplied by the relative abundance of the species, then adds the results of all species. Higher values of the Shannon index indicate higher diversity of the community. The calculation formula is:

(4) $$H=-\sum_{i=1}^{S}\left(P_{i}\ln P_{i}\right)$$

where S was the number of species in the sample plot, and Pi was calculated as follows: Pi = Ni/N, where Ni represents the number of individuals of species i and N represents the total number of individuals of all species in one particular plot, respectively.

Simpson is similar to the Shannon index. It also considers the richness and evenness of species, but it focuses more on the relative abundance of species. The closer the value of the Simpson index is to 1, the higher the diversity of the community. The calculation formula is:

(5) $$Cd=1-\sum_{i=1}^{S}{\left(P_{i}\right)}^{2}$$

Considering only the number of species and the total number of individuals, and the number of species in a sample of a certain size is defined as the Margalef diversity index. The calculation formula is:

(6) $$D=\frac{S-1}{\ln N}$$

Pielou reflects the degree of uniform distribution of the functional types. When the value of Pielou’s index is larger, it represents higher species homogeneity, i.e., a more even distribution of abundance across species; when the value of the index is close to 0, it indicates that some species dominate in abundance and few others. The calculation formula is:

(7) J=H/ln(S)

Chao 1 uses an algorithm to estimate an index of the number of ASVs contained in a sample, estimating the number of species actually present in a community by counting the number of ASVs detected only once and twice in the community. Thus, when the Chao 1 index is large, it suggests that more species may be present in the community that have not yet been observed, thus indicating higher species diversity. The calculation formula is:

(8) $$Chao1=S_{obs}+\frac{F_{1}(F_{1}-1)}{{2(F}_{2}+1)}$$

where $S_{obs}$ is the observed number of species, and $F_{1}$ and $F_{2}$ are the number of species with abundances of 1 and 2.

The stability of microbial communities is primarily attributed to species diversity. This study employed the Average Variability Degree (AVD) index, as used by Niu et al. [29], to assess microbial community stability. Usually, the lower AVD represents higher stability of the community. Although this index is not a classical measure of stability, it is widely used in microbial ecology research to characterize the degree of dispersion or variability within a community. The AVD was calculated as follows:

(9) $$AVD=\frac{\sum_{i=1}^{n}\frac{\left|x_{i}-{\bar{x}}_{i}\right|}{\delta_{i}}}{k\times n}$$

where k is the number of samples (if the AVD of a single sample is sought, k is 1; if the AVD of a group of samples, k is the number of samples of that group) and n is the number of OTU. And $x_{i}$ is the abundance of OTUi in each sample, and $\bar{x_{i}}$ and $\delta_{i}$ are the mean and standard deviation of OTUi abundance in all samples, respectively.

We constructed interaction networks for microorganisms, including bacteria and fungi, from different land use analysis treatments in the WFLZ. To construct the overall coexistence network of OTUs, only microorganisms with relative abundances ≥ 0.1% were retained for subsequent analysis. Spearman correlation coefficients between OTUs were calculated, and weakly correlated or non-significant relationships (|R| < 0.7 or *p* > 0.05) were excluded (assigned a value of 0). Based on the adjacency list generated from this adjacency matrix, a network diagram was constructed using Gephi software 0.10.1 (Developed by ATRIUM (Advanced Technology Research Institute of the University of Maine), a French research institution.), and five topological metrics were calculated (modularity class, clustering coefficient, nodes, edges, and degree). The interaction networks were all visualized in Gephi using a force-guided layout algorithm with 104 permutations, and to ensure that the resulting empirical networks were non-random and non-scale, they were evaluated in comparison to random networks with the same number of nodes and edges [18,26].

### 2.4. Data Analysis and Software

#### 2.4.1. Data Analysis

Data analysis was performed using SPSS 25.0 and R 4.5.1. All measured data were confirmed to follow a normal distribution and passed the test for homogeneity of variance. One-way analysis of variance (one-way ANOVA) followed by Duncan’s multiple comparisons test (α = 0.05) was applied to assess the significance of differences in soil nutrients, enzyme activities, and α- and β-diversity indices. Differential microorganisms across communities were identified using Linear Discriminant Analysis Effect Size (LEfSe) [15,39]. To explore the relationships between environmental variables and microbial communities, two multivariate techniques were employed. Canonical Correlation Analysis (CCA) was used to reflect the overall correlation between two sets of indicators (e.g., soil properties and microbial community composition) by maximizing the correlation between their linear combinations. The Mantel test was further applied to examine the correlation between a community distance matrix (e.g., Bray–Curtis) and environmental distance matrices (e.g., based on pH, temperature). A larger Mantel correlation coefficient with a smaller *p*-value indicates a stronger influence of environmental factors on the microbial community structure [34,35]. Finally, a mediation model was constructed to analyze the significant pathways through which land use affects different microbial groups. Model fit was evaluated using four statistical indicators: CFI, chi-square, Standardized Root Mean Square Residual (SRMR), Root Mean Square Error Approximation (RMSEA), and *p*-value (*p* > 0.05, the model fits well). A better model fit is indicated by smaller values for Fisher’s C, chi-square, and AIC [31,35].

#### 2.4.2. Software

All graphical outputs were generated using R 4.5.1. The primary R packages and their applications are as follows: dplyr and tidyr were used for data preprocessing; phyloseq was employed to handle biological data objects and calculate alpha diversity indices; vegan was utilized to compute beta diversity (Bray–Curtis distance), perform PERMANOVA analysis (adonis2 function), and conduct related ecological statistics. SpiecEasi or igraph is used to construct microbial co-occurrence networks and compute network topological features (e.g., specific packages and methods used are detailed). Lavaan was employed for fitting structural equation models and calculating goodness-of-fit indices. Stats was used for analysis of variance (ANOVA) and post hoc tests; lmtest or car packages were used to test for homogeneity of variance. Ggplot2 was used to generate all core graphics; ggpubr or cowplot were used for graphical layout.

## 3. Result

### 3.1. Soil Nutrients and Enzyme

**Figure 2:**
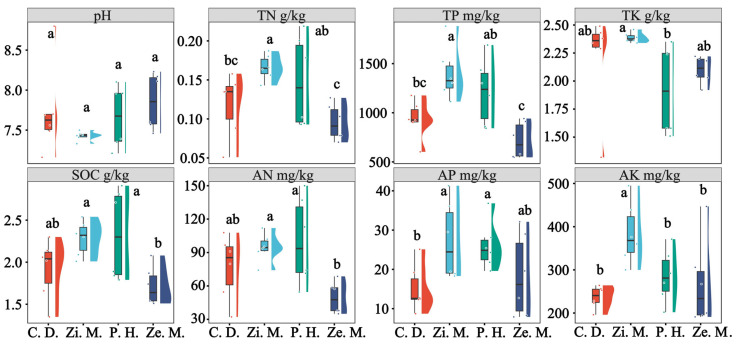
The difference in soil physical and chemical among four types of different plant communities in the WLFZ. SOC: soil organic carbon; TN: total nitrogen; TP: total phosphorus; TK: total potassium; AN: alkaline hydrolyzable nitrogen; AP: available phosphorus; AK: available potassium. Notes: Zi. M.: *Ziziphus mauritiana*; C. D.: *Cynodon dactylon*; P. H.: *Parthenium hysterophorus*; Ze. M.: *Zea mays*; the same below. The lowercase letters indicate significance at the *p* = 0.05 level.

The soil chemical of four different plant communities in the WLFZ significantly changed (*p* < 0.05), especially SOC, N, P, and K (Figure 2). Notably, there was a similarly changed trend that included TN, TP, SOC, TK, AN, AP, and AK (*p* < 0.05). However, pH insignificantly changed (*p* > 0.05). Compared to the *Z. mauritiana* treatment, the pH slightly increased by 4.13%, 3.16% and 5.79% in the *C. dactylon*, *P. hysterophorus* and *Z. mays* treatments, respectively, whereas TN, SOC, TP and AN noticeably showed a downward trend in the *Z. mays*, *P. hysterophorus* and *Z. mauritiana* treatments. Similarly, relative to the *Z. mauritiana* treatment, AP significantly reduced by 44.57%, 4.51% and 32.89% in the C. dactylon, *P. hysterophorus* and *Z. mays* treatments, respectively, and AK collapsed by 38.38%, 25.72% and 30.37% in the *C. dactylon*, *P. hysterophorus* and *Z. mays* treatments, respectively. In conclusion, TN, TP, SOC, AN, AP and AK attained their peak values, and pH reached its minimum values in the *Z. mauritiana* and *P. hysterophorus* treatments. Because of the pH of maximum values in the *Z. mays* treatment, its soil chemical decreased to the bottom, especially TN, TP, SOC and AN.

**Figure 3:**
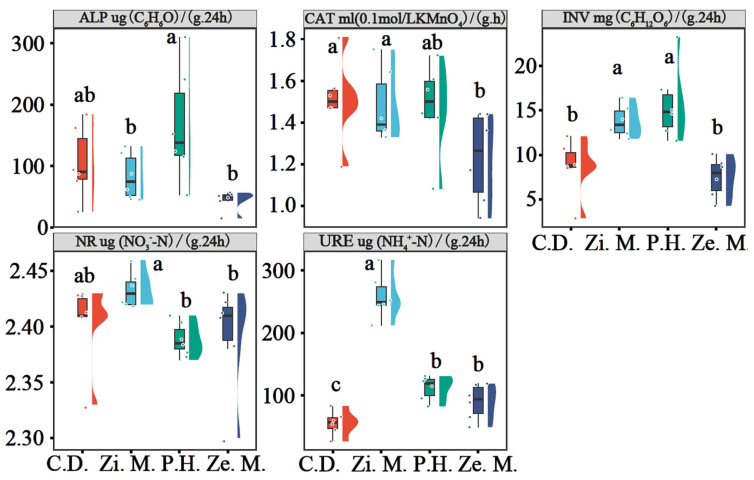
The difference in soil enzymes among four types of different plant communities in the WLFZ. URE: Urease; INV: Sucrase; NR: Nitrate reductase; CAT: Catalase; ALP: Alkaline phosphatase. The lowercase letters indicate significance at the *p* = 0.05 level.

In the WLFZ, five enzyme activities of four different plant communities showed a significant variation (*p* < 0.05), which were their minimum values in the *Z. mays* treatment and their maximum values with slight fluctuations in the *Z. mauritiana* and *P. hysterophorus* treatments (Figure 3). As shown in Figure 3, there was a sharply decreasing trend of the ALP, which decreased by 46.12% with the *Z. mays* treatments, whereas the *P. hysterophorus* and *C. dactylon* treatment of the ALP rapidly picked up by 100.63% and 26.57%, respectively, compared with the *Z. mauritiana* treatment. There was a similar trend in which CAT declined steadily by −1.88%, 0.31% and 16.61%, separately, and URE suddenly plummeted by 78.51%, 56.52% and 65.25% in the *C. dactylon*, *P. hysterophorus* and *Z. mays* treatments compared with the *Z. mauritiana* treatment. Additionally, INV reached the peak at 15.73 mg/(g·24 h) and NR attended the bottom at 2.38 μg/(g·24 h) in the *P. hysterophorus* treatment. In conclusion, the URE, NR and CAT of the *Z. mauritiana* treatment was significantly higher than other treatments, and the INV and ALP of *P. hysterophorus* treatment had an increasing trend and those of the *Z. mays* and *C. dactylon* treatments had a descending tendency.

### 3.2. Composition and Abundance of the Soil Bacterial and Fungal Community

**Figure 4:**
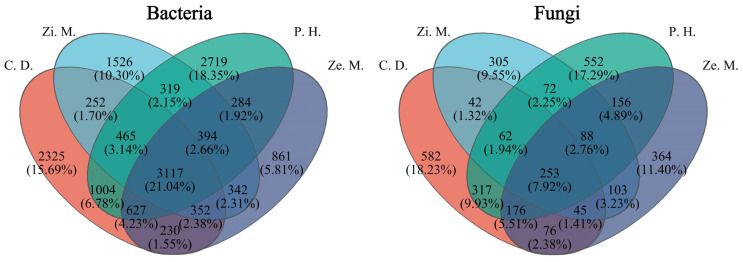
Venn diagram of common and endemic species of soil bacteria and fungi under four types of different communities.

To clarify the composition and abundance of the soil bacteria and fungi communities, we used a Venn diagram that distinguishes common and endemic species of soil bacteria and fungi under different communities of four types (Figure 4). For the bacteria community, soil samples under different communities of four types were acquired; the average number of sequences was 62,707, the total of OTUs was 74,955 and the rate of covering was from 99.99% to 100%, which demonstrated the rational sequencing data and also reflected species and fundamental structure of the soil bacteria community. The common OTU of soil bacteria was 3117 under different communities of four types in the WLFZ. Meanwhile, there was a significant difference in the endemic OUT of different communities, of which the *P. hysterophorus* treatment had the highest number of OUTs with 2719, followed by the *C. dactylon* treatment with 2325, closely followed by the *Z. mauritiana* treatment with 1526, and the *Z. mays* treatment had the lowest number of OUTs with 861.

For the fungi community, soil samples under different communities of four types were acquired with the average number of sequences 72,252, a total of OTUs 12,053 and the rate of fungi covering from 99.99% to 100%, which demonstrated the rational sequencing data and also reflected species and fundamental structure of soil fungi community. The common OTU of soil fungi was 253 under different communities of four types in the WLFZ. Meanwhile, there was a significant difference in the endemic OUT of four different communities, with the highest number of endemic OUT to the *C. dactylon* treatment at 582, followed by the *P. hysterophorus* treatment at 552, close behind the *Z. mays* treatment at 364, and the lowest number of endemic fungi OUT to the *Z. mauritiana* treatment at 305.

**Figure 5:**
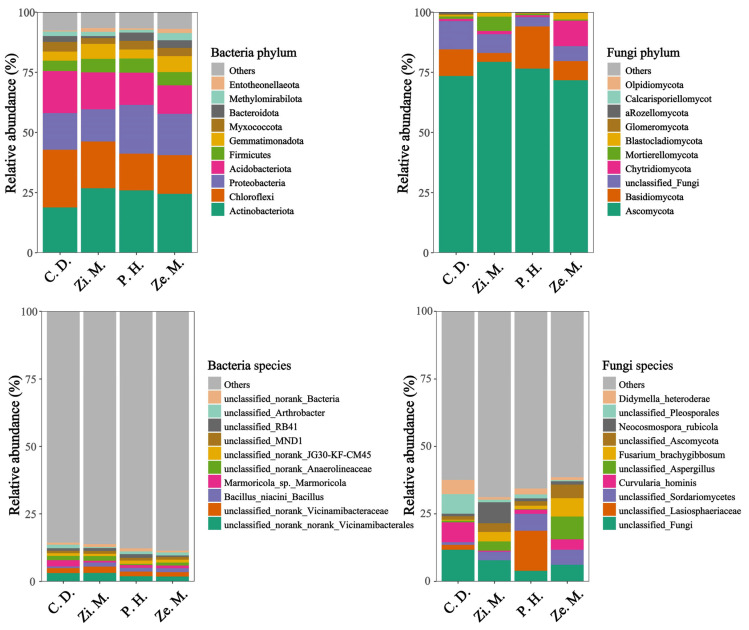
Relative abundance of soil bacteria and fungi at the phylum and species level under four types of different communities.

To analyze the phylum and species of soil bacteria and fungi communities, we chose the 10 dominant phylum and species to analyze differences in the different communities of 4 types. According to Figure 5, at the phylum classification level, the important phylum of bacteria included *Actinobacteriota* between 18.73% and 26.70%, *Chloroflexi* between 15.46 and 24.02%, *Proteobacteria* between 13.28% and 20.04%, *Acidobacteriota* between 11.64% and 17.52%, *Firmicutes* between 4.30% and 5.73%, *Gemmatimonadetes* between 3.81% and 6.56%, *Myxomycota* between 2.43% and 3.98%, *Bacteroidetes* between 0.93% and 3.47%, of which these phylum accounted for the total of the bacteria community from 88.28% to 91.48%. The *Proteobacteria* relative abundance of the *P. hysterophorus* treatment was stably higher than the remaining three communities, and the *Acidobacteriota* and *Chloroflexi* relative abundance of the *C. dactylon* treatment was noticeably higher than the remaining three communities. The vital phylum of fungi contained *Ascomycota* within a small range of 71.08–78.79%, *Basidiomycota* within a large range of 3.76–16.81%, *unclassified_k_Fungi* within a small range of 3.92–9.77%, *Chytridiomycota* within a large range of 0.69–11.58%, *Mortierellomycota* within a small range of 0.57–6.45%, of which these phylum represented the total of bacteria community from 97.01% to 99.30%. The *Basidiomycota* relative abundance of the *P. hysterophorus* treatment was overwhelmingly higher than the other treatments, the *unclassified_Fungi* relative abundance of the *C. dactylon* treatment was significantly higher than the other treatments, and the *Chytridiomycota* relative abundance of the *Z. mays* treatment was markedly higher than the other treatments. Similarly, the *Mortierellomycota* and *Ascomycota* relative abundance of *Z. mauritiana* treatment was considerably higher than the other treatments; specifically, the content of *Ascomycota* was highest and its relative abundance occupied 78.79% in the total of fungi communities.

At the species level, there were many unclassified species and little-classified species, of which the species of bacteria was only two species were distinguished, *Marmoricola* and *Bacillus,* and those of fungi were only divided to four species, *Curvularia*, *Fusarium*, *Neocosmospora*, and *Didymella*. The *Marmoricola* was higher and the *Bacillus* was lower in the *C. dactylon* treatment than the other treatments. Similarly, the *Curvularia* and *Didymella* of the *C. dactylon* treatment, the *Fusarium* of the *Z. mays* treatment and the *Neocosmospora* of the *Z. mauritiana* treatment were markedly higher than the other treatments, respectively.

**Figure 6:**
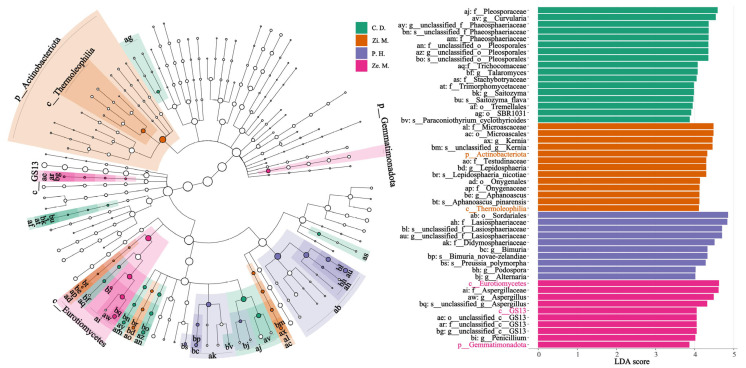
LEfSe analysis of bacteria and fungi in four types of different communities.

Because of having many unclassified species, we utilized the linear discriminant analysis Effect Size (LEfSe) to estimate the magnitude of the abundance and species on the differential communities and combined this with the species evolutionary branching diagram to show the differential species and their evolutionary relationship. We used the LEfSe to display the 50 most significantly featured species and the 200 most abundant taxa in Figure 6, of which there was a different trend in the bacteria and fungi that the significant species of the fungi was noticeably higher than those of bacteria. It was worth noting that the bacterial biomarkers only explained the order, but the fungal biomarkers could explain the species. In the difference of the four types, the abundance of bacterial biomarkers were slightly higher in the *Z. mauritiana* treatment than other treatments, and the abundance of fungal biomarkers were slightly higher in the *C. dactylon* treatment other treatments. At the same time, the *C. dactylon*, *Z. mauritiana*, and *Z. mays* treatments had both the bacterial and fungal biomarkers, whereas the *P. hysterophorus* treatment was only one of the fungal biomarkers.

### 3.3. Diversity Index of Soil Bacteria and Fungi

To illustrate microbe diversity, we utilized α and β diversity, PCoA and Anosim analysis to calculate. As shown in Table 3, bacterial diversity significantly changed under four types of different communities in the WLFZ (*p* < 0.05). Meanwhile, the six types of bacterial diversity of the *P. hysterophorus* treatment were markedly higher and those of the *Z. mays* treatment were noticeably lower than under other treatments, respectively. Of course, the richness, Margalef and Chao 1 of fungal diversity of the *P. hysterophorus* treatment was markedly higher and those of the *Z. mauritiana* and *Z. mays* treatments were noticeably lower than other treatments, respectively. However, the Shannon–Wiener, Simpson and Pielou index of fungal diversity insignificantly changed under four types of different communities in the WLFZ (*p* > 0.05), but those of the *C. dactylon* treatment was higher than other treatments.

**Figure 7:**
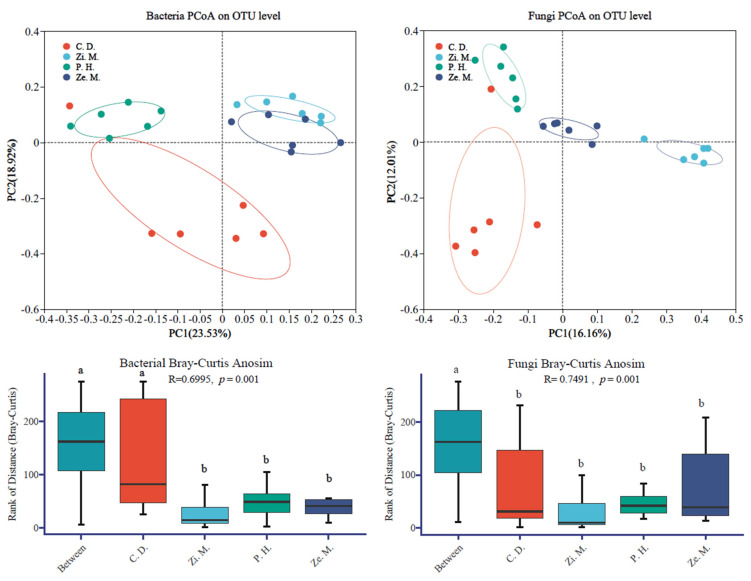
Principal co-ordinate analysis (PCoA) and Anosim analysis of soil bacterial and fungi communities under different community types. The lowercase letters indicate significance at the *p* = 0.05 level.

Similarly, at the OTU level, the Bray–Curtis algorithm was utilized for the principal co-ordinates analysis (PCoA) to analyze beta diversity of the bacteria and fungi (Figure 7). PCoA could reflect the difference in bacteria and fungi among the different communities of four types on the WLFZ, for which the two-dimensional sorting plot of PCoA could distinguish the sample of different communities types using different colors, and the PC1 and PC2 was the most dominant characteristic value to explain the analyzed result of bacteria and fungi, respectively.

The bacterial result of PCoA showed that the different communities structures of the four types were without overlap and obviously markedly distinctions, while the partial structures of *Z. mauritiana* and *Z. mays* communities were overlapped. Meanwhile, the contribution rate of PC1 and PC2 occupied 23.53% and 18.95%, respectively, and the contribution rate of both was at 42.45%, which indicated that the bacterial community of different community types had a highly noticeable difference; especially the community structure of bacteria was significantly different between *P. hysterophorus* and *C. dactylon* treatment. At the same time, in the Anosim analysis result, the R was 0.6995 and the *p* was 0.001, which could reveal that the distance between groups was dramatically greater than the within-group distance.

The fungal result of PCoA and Anosim analysis was similar to the bacterial result in that the different communities structure of four types were without overlap and obviously markedly distinction, and the distance between groups was dramatically greater than the within-group distance. Among them, the fungal PCoA figure demonstrated that the contribution rate of PC1 and PC2 occupied 16.16% and 12.01%, respectively, and the contribution rate of both was at 28.17%, and the Anosim result of R and *p* was 0.7941 and 0.001, respectively.

### 3.4. Effect of Different Communities on Microbial Community Stability and Network Patterns

**Figure 8:**
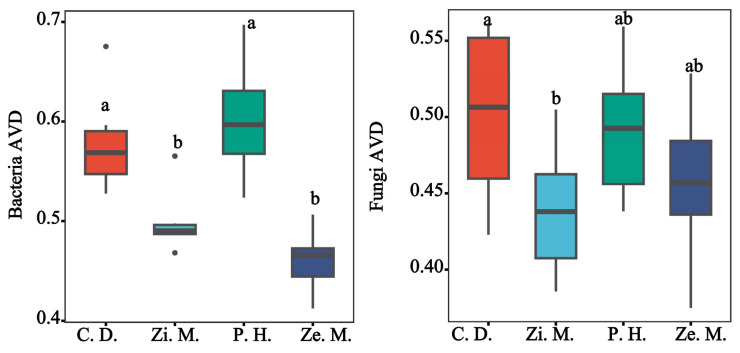
Community stability visualization of bacteria and fungi among the different communities of four types by the WLFZ. The lowercase letters indicate significance at the *p* = 0.05 level.

The bacterial and fungal community stability were evaluated on the AVD index in the different communities of four types where the lower of AVD represented the stably higher microbial community. As shown in Figure 8, the AVD of bacteria and fungi markedly changed (*p* < 0.05), the AVD of bacteria was the peak at 0.6 on the *P. hysterophorus* treatment and the fungal AVD reached the top at 0.5 on the *C. dactylon* treatment, which gave specific information about the low community stability in the bacteria of *P. hysterophorus* treatment and fungi of *C. dactylon* treatment. Among them, the AVD of bacteria and fungi always maintained the lowest point on the *Z. mauritiana* and *Z. mays* treatment, which showed that the *Z. mauritiana* and *Z. mays* treatment possessed the highest community stability.

**Figure 9:**
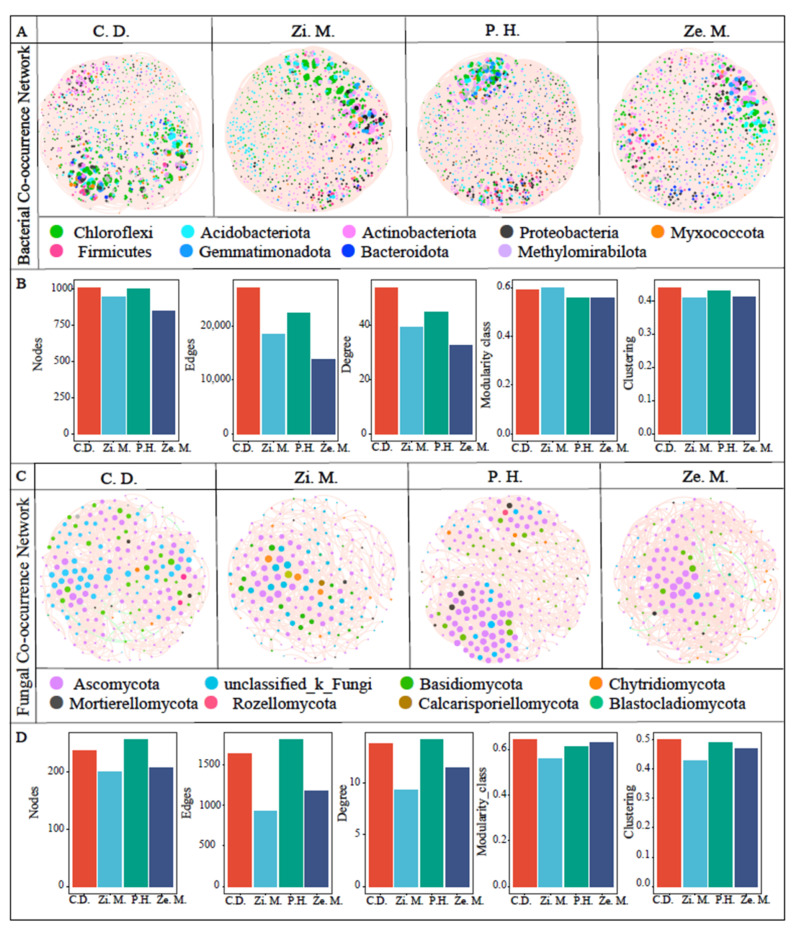
Co-occurrence network visualization of bacterial and fungal patterns among the different communities of four types in the WLFZ (**A**,**C**). The bacterial and fungal co-occurrence patterns included five network topologies, such as nodes, edges, degree, modularity class and clustering (**B**,**D**).

The bacterial and fungal co-occurrence network patterns presented a significant difference based on the phylum level. As shown in Figure 9A,B, the bacterial community structure of the *C. dactylon* treatment was the most complicated, followed by the *Z. mauritiana* and *P. hysterophorus* treatments, and finally the *Z. mays* treatment. Among them, the first four dominant phyla were *Chloroflexi*, *Acidobacteriota*, *Actinobacteriota* and *Proteobacteria* on the *C. dactylon* and *Z. mauritiana* treatments, which separately accounted for their total amounts at 27.3%, 16.72%, 15.92%, 14.94% and 24.47%, 22.35%, 15.57%, 11.65%. Meanwhile, the top four dominant phyla in the *P. hysterophorus* and *Z. mays* treatments were *Actinobacteriota*, *Chloroflexi*, *Proteobacteria* and *Acidobacteriota*, which accounted for 22.24%, 19.18%, 15.65%, 12.12% and 22.82%, 19.22%, 18.52%, 14.91%, respectively. The nodes, edges, degree, modularity class and clustering also indicated that the network topology of *C. dactylon* treatment was more complex than the other treatments. As shown in Figure 9C,D, the fungal co-occurrence network of *P. hysterophorus* and *C. dactylon* treatments were more complicated than *Z. mauritiana* and *Z. mays* treatments, which the nodes, edges, degree, modularity class and clustering also indicated. It is noteworthy that the first three dominant phyla of the four different communities were *Ascomycota*, *unclassified_k_Fungi* and *Basidiomycota*, which accounted for 56.36%, 24.58%, 13.14% on the *C. dactylon* treatment, and 52.76%, 26.63%, 10.05% on the *Z. mauritiana* treatment, and 74.51%, 7.84%, 10.98% on the *P. hysterophorus* treatment, and 65.53%, 11.65%, 15.05%, respectively.

### 3.5. Using CCA and Mantel Test Analyze the Effect of Soil Nutrient and Enzyme Activity with Structure of Community and Diversity

**Figure 10:**
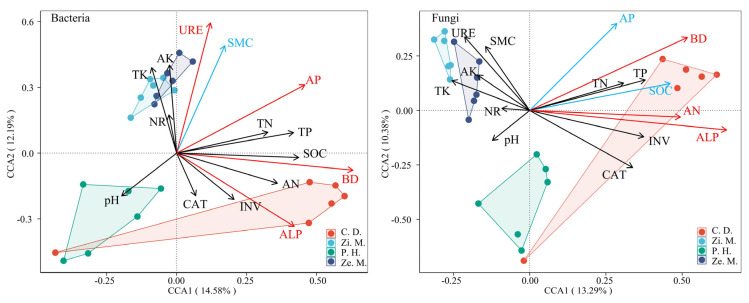
The CCA reflected correlation relationship of soil nutrient and enzyme activity with microbial community structure. The red arrow represents that the significant values were < 0.05, the blue arrow represents that the significant values were >0.05 and <0.1, and the black arrow represents that the significant values were >0.1. Notes: BD: Soil bulk density; SMC: Soil moisture content; SOC: soil organic carbon; TN: total nitrogen; TP: total phosphorus; TK: total potassium; AN: alkaline hydrolyzable nitrogen; AP: available phosphorus; AK: available potassium; URE: urease; INV: sucrase; NR: nitrate reductase; CAT: catalase; ALP: alkaline phosphatase.

The CCA revealed the relative effects of soil factors on bacteria and fungi (Figure 10). The soil factors could only explain 26.77% (CCA1 = 14.58%, CCA2 = 12.19%) and 23.67% (CCA1 = 13.29%, CCA2 = 10.38%) of the bacteria and fungi community, respectively. The result indicated that the URE, AP, BD and ALP held a significant correlation relationship with the bacterial community of four different communities, and the BD, AN and ALP had a marked correlation with fungi of different communities (*p* < 0.05).

**Figure 11:**
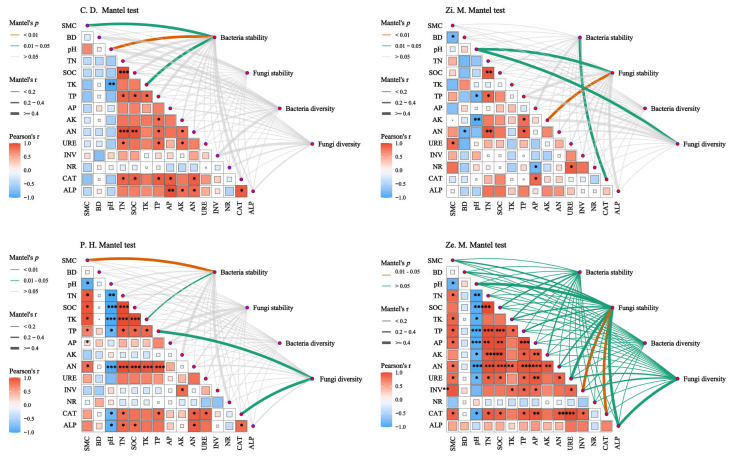
Correlation analysis between soil factors and microbial diversity and stability among different communities of four types in the WLFZ. The color gradient of the half-matrix in the figure represents the Pearson’s correlation coefficient, *** indicates *p* < 0.001; ** represents *p* < 0.01; * represents *p* < 0.05. The width of the line represents the Mantel’s r statistic corresponding to the distance correlation, and the color of the line represents the statistical significance indicated by Mantel’s *p*. Notes: BD: Soil bulk density; SMC: Soil moisture content; SOC: soil organic carbon; TN: total nitrogen; TP: total phosphorus; TK: total potassium; AN: alkaline hydrolyzable nitrogen; AP: available phosphorus; AK: available potassium; URE: urease; INV: sucrase; NR: nitrate reductase; CAT: catalase; ALP: alkaline phosphatase.

As shown in Figure 11, the results of correlation analysis showed that soil factors apart from BD, SOC, TN, ALP, URE and NR had a highly correlational relationship, including SMC, pH, TK, TP, AP, AK, INV, and CAT in the four different communities. Notably, SMC, pH, and TK were important factors and markedly affected the bacterial community in the *C. dactylon* community; pH and AK significantly affected fungal community stability and diversity, and CAT affected bacterial stability in the *Z. mauritiana* community; SMC and TK were crucial factors for bacterial stability, and TP and CAT markedly affected fungal diversity in the *P. hysterophorus* community. Conversely, the fungal stability in the *Z. mays* community was correlated with INV and CAT. Findings indicate that SMC and pH exert crucial roles across all four distinct communities.

### 3.6. Used Intermediary Analysis Explores the Fungal and Bacterial Influence Factors Among the Four Different Communities in the WLFZ

**Figure 12:**
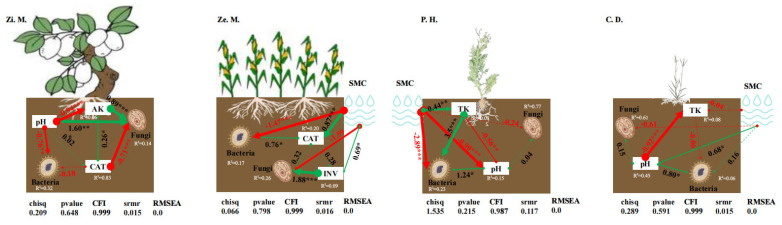
Correlation analysis between soil factors and microbial diversity and stability among the different communities of four types in the WLFZ. Green arrows represent significant positive paths, red arrows represent significant negative paths; dashed arrows indicate non-significant paths, and * of the path coefficients of the arrows indicate *** *p* < 0.001, ** *p* < 0.01, and * *p* < 0.05, respectively. Notes: SMC: Soil moisture content; TK: total potassium; AK: available potassium; INV: sucrase; CAT: catalase.

To avoid using too many factors to establish intermediary analysis, we utilized CCA and Mantel test to choose some significant effects which explained bacterial and fungal community stability among the different communities of four types in the WLFZ. Therefore, we selected some significant factors (as observed variables) which were soil properties which included SMC, pH, AK, and TK, and soil enzymes which contained INV and CAT, to establish intermediary analysis which explained bacterial and fungal community stability (as the dependent variable) under the four different communities (Figure 12). The results of intermediary analysis revealed distinct response pathways of bacterial and fungal community stability in the four different communities (*p* > 0.05) (Figure 12). Specifically, in the *Z. mauritiana* community, soil pH served as a primary variable significantly affecting the stability of both bacterial and fungal communities, with path coefficients of 1.6 and −0.76, respectively. AK regulated fungal community stability indirectly through a mechanism dependent on the synergistic interaction between pH and CAT, with path coefficients of −0.94 and 0.26. Conversely, CAT exhibited a significant negative effect on fungal community stability (path coefficient = −0.71). In the *Z. mays* community, soil moisture content in the WLFZ significantly influenced the stability of bacterial and fungal communities, with path coefficients of −1.47 and −1.59, respectively. Bacterial community stability was indirectly affected by CAT, whereas fungal community stability was positively and significantly influenced by INV through an indirect pathway. Specifically, soil moisture content showed a strong positive effect on CAT (path coefficient = 0.87, *p* < 0.001), which was greater than the effect of CAT on bacterial community stability (path coefficient = 0.76, *p* < 0.05). Similarly, the positive effect of INV on fungal community stability (path coefficient = 1.88, *p* < 0.001) was significantly stronger than that of soil moisture on INV (path coefficient = 0.69, *p* < 0.05). In the *P. hysterophorus* community, bacterial community stability was significantly influenced by soil moisture content, TK, and pH. Soil moisture content showed a negative correlation with both bacterial community stability and pH, whereas TK and pH positively and significantly enhanced bacterial community stability, with path coefficients of 3.5 and 1.24, respectively. In the *C. dactylon* community, pH modulated bacterial community stability through an indirect pathway. Notably, only soil moisture content significantly affected bacterial community stability by altering soil pH, with path coefficients of 0.68 and 0.8, respectively.

## 4. Discussion

### 4.1. Effect of Soil Nutrients and Enzyme Among the Different Communities of Four Types in the WLFZ

The WLFZ is a transition zone between water and land, where a complex process of ‘flooding–drying’ occurs over time [40,41]. This process also noticeably affects soil physical and chemical properties, changes in enzyme activities and microbial communities [27,42]. Numerous studies have shown that seasonal wet and dry alteration of WFLZ significantly affects the farming practices of the neighboring residents and the survival of the wild plant communities due to the change in water level, which in turn significantly alters the physical and chemical properties and enzyme activities of the WFLZ [24,43]. In this study, however, we observed that the invasive plant *P. hysterophorus* significantly reduced soil BD during early flooding stages, while the SMC in *Z. mauritiana* treatment markedly increased as a result of local irrigation practices (*p* < 0.05). A similar phenomenon has been reported in the Three Gorges Reservoir area, where soil BD increased due to physical erosion and acidity neutralization [8,9]. Interestingly, the pH in *Z. mauritiana* treatments was lower than that in *P. hysterophorus*-invaded plots. This discrepancy may be attributed to a combination of agricultural activities and the intrinsic growth traits of *Z. mauritiana* [17,44]. On one hand, tillage and chemical fertilizer application tend to lower soil pH [45]. On the other hand, the well-developed root system of *Z. mauritiana* helps to loosen soil structure after irrigation [46]. Notably, BD, SMC, and pH collectively function as dominant parameters governing soil properties, and their dynamic variations influence the physiological activity of the *Z. mauritiana* community [47,48]. Previous research suggests that densely distributed invasive plants can increase soil porosity and reduce BD [42,49]. Consistent with this, our findings indicate that the invasive plant of *P. hysterophorus* secretes low-molecular-weight organic acids from its root system, enhancing nutrient solubilization and sequestration to support its vigorous growth and rapid reproduction [10,26].

Soil elements inevitably undergo migration and transformation following the alternating “‘flooding–drying” cycles in the WLFZ [46,50]. Generally, physical and chemical properties of the soils in this area are closely interrelated [6,9]. Previous studies have indicated that elevated pH is often associated with increased N content in WLFZ soils [11]; however, some reports also suggest that higher soil bulk density (BD) corresponds to greater soil nutrient levels [12]. The present study revealed that the *P. hysterophorus* site exhibited relatively high pH and BD, along with the highest contents of SOC, N, and P. In contrast, although the *Z. mays* site had a higher pH than *P. hysterophorus*, its BD was lower, and it displayed the lowest soil nutrient content. These findings imply that the invasive plant *P. hysterophorus* demonstrates a strong colonization capacity in the WLFZ, whereas cultivating *Z. mays* may contribute to soil nutrient loss in this area [51,52]. Furthermore, significant differences in soil nutrients were observed under different land-use types [10,46]. With the exception of potassium (K), the soil nutrient levels under *P. hysterophorus* were comparable to those under the crop *Z. mauritiana*, further supporting the adaptability of *P. hysterophorus* in the WLFZ environment [53]. In comparison, the native plant *C. dactylon* showed lower contents of C, N, and P than both the invasive *P. hysterophorus* and the cropland *Z. mauritiana*, highlighting distinct soil nutrient utilization strategies among different vegetation types in the typical WLFZ of the Jinsha River Basin [54,55,56].

Soil enzymes act as catalysts in soil nutrient cycling, and their activities serve as reliable indicators of soil fertility and microbial communities, reflecting key processes such as accumulation, decomposition, and mineralization of soil nutrients [13,57]. Generally, soil enzyme activities are regulated by microbial communities structure and soil physicochemical properties, while different plant communities can further modulate enzyme activities through their influences on soil nutrient availability and microbial composition [21,47]. Consistent with the findings of prior research, which reported significant differences in soil enzyme activities among different vegetation types [58], our study also observed notable variations across plant communities. Specifically, the invasive species of *P. hysterophorus* showed significantly lower activities of urease and nitrate reductase compared to the other three plant communities, but higher activities of alkaline phosphatase, catalase, and invertase.

It is noteworthy that soil invertase, urease, and phosphatase are closely associated with the transformations of carbon, nitrogen, and phosphorus in the soil, respectively [22,59]. In this study, the *Z. mays* consistently exhibited the lowest enzyme activities, aligning with its relatively poor soil nutrient status [56,60]. In contrast, the enzyme activities of the *P. hysterophorus* did not differ significantly from those of the *Z. mauritiana*, suggesting that the *P. hysterophorus* can rapidly adapt to the fluctuating hydrological conditions in the WLFZ and enhance soil nutrient cycling through its own enzymatic secretions, thereby facilitating its establishment and growth [54,61]. These results demonstrate that the invasive plant of *P. hysterophorus* can effectively increase productivity through positive feedbacks in nutrient cycling, leading to higher levels of soil nutrients and enzyme activities [53,59,62].

### 4.2. Composition and Diversity of the Soil Microbe

Microorganisms represent a core component of soil ecosystems, serving as key drivers of material cycling and energy flow, and playing an indispensable role in maintaining the structural and functional stability of soil ecosystems [5,60]. According to prior studies, the composition and structure of microbial communities significantly influence soil nutrient cycling and the ecosystem multifunctionality [58,63]. The results of this study demonstrate that the relative abundances of both bacterial and fungal communities were significantly higher in areas dominated by the invasive plant *P. hysterophorus* and the native vegetation *C. dactylon* compared to *Z. mays* and *Z. mauritiana*, indicating that plant community type exerts a substantial influence on microbial assemblage [4,57,61]. These differences are likely associated with variations in plant diversity and soil nutrient cycling processes across vegetation types [46,50]. Specifically, the relative abundance of *Proteobacteria* was significantly elevated in the *P. hysterophorus* community compared to other treatments, whereas *Chloroflexi* and *Acidobacteriota* were more abundant in the *C. dactylon* community. This divergent distribution may be attributed to differential regulation of environmental factors (such as BD, moisture, pH, and SOC) by the distinct vegetation types [64,65]. Notably, *Proteobacteria* are generally associated with nutrient-rich soil conditions, and their prevalence provides microbial evidence supporting the observed higher soil nutrient levels in *P. hysterophorus* community. In contrast, *Acidobacteriota* are often adapted to nutrient-poor environments, which is consistent with the lower nutrient status observed under *C. dactylon* community [22,34].

Additionally, this study employed LEfSe to identify biomarkers across different samples. Results indicate that bacterial biomarkers could only be annotated to the order level, whereas fungal biomarkers could be annotated to the species level (Figure 6). This discrepancy likely stems primarily from the fact that while the widely used bacterial 16S rRNA gene database offers broad coverage, its resolution at the species level is limited [29,30]. Many environmental bacterial OTUs are difficult to accurately align to the species level, and the 16S rRNA gene is sometimes overly conserved between species, making it challenging to distinguish closely related species [22,36]. In contrast, fungal ITS databases (e.g., UNITE) provide richer and more comprehensive annotation information at the species level [34,37]. The ITS region exhibits faster evolutionary rates, offering superior interspecies resolution. This limitation implies that discussions regarding bacterial community function in this study primarily remain at higher taxonomic levels (order level) [16,45]. While changes at the order level can partially reflect shifts in ecological function, they may obscure niche differentiation among species within the same order [36,37]. Therefore, caution should be exercised when interpreting the ecological significance of bacterial markers.

Different plant communities shape distinct soil microbial abundance, which in turn influences soil enzyme activities and the transformation capacity of SOC, ultimately affecting soil fertility under the vegetation [29,56]. According to some studies, an increase in ammonium nitrate content in soil nutrients can alter soil microbial abundance, thereby enhancing soil enzyme activity and soil fertility [13,59]. In the present study, the diversity indices of the invasive plant of *P. hysterophorus* and the native vegetation of *C. dactylon* were significantly higher than those of the *Z. mays* and *Z. mauritiana* (*p* < 0.05). One study had shown that higher plant diversity promotes the evolution of microbial functional groups [16]. Our findings indicate that the lower microbial diversity in *Z. mays* and *Z. mauritiana* is not only related to soil nutrients but also associated with the composition of the plant community [45,52]. In addition, farming management practices applied by local residents to *Z. mays* and *Z. mauritiana* significantly affect soil nutrients and enzyme activities, thereby fostering beneficial microbial communities that support plant growth and development [57,58]. This corresponds with the observed lower microbial diversity and network complexity, along with higher community stability, in *Z. mays* and *Z. mauritiana* in this study [55,60]. Furthermore, beta diversity analysis confirmed that microbial diversity and community structure differ significantly across vegetation types, with between-group differences being substantially greater than within-group differences (*p* < 0.05) (Figure 7). This is consistent with previous findings, which indicated that different vegetation types create distinct soil environments that drive microbial community variation [31]. Previous studies have also suggested that richer plant composition intensifies inter-specific competition [8,66], particularly when the invasive plant *P. hysterophorus* invades native plant communities, leading to more intense competition for soil nutrients and microbial resources [5,14]. This aligns with the conclusions of our study, which found that the bacterial and fungal AVD of the invasive plant of *P. hysterophorus* was slightly higher than that of the native plant of *C. dactylon*, indicating that the establishment of the *P. hysterophorus* in the WFLZ significantly enhances soil nutrients and enzyme activities, thereby reducing the stability of the microbial community [51,54,64].

The stability of microbial communities is mainly attributed to species diversity, as it is usually considered to have a positive effect on the stability of microbial communities, whereas loss of species usually leads to impaired ecosystem functioning [15,60]. Generally speaking, higher plant diversity is associated with richer functional groups in microbial communities and more intense niche competition, which consequently leads to higher soil microbial diversity, greater co-occurrence network complexity, and reduced stability of the microbial community [16,66]. These patterns are consistent with the results observed in our study for the invasive plant of *P. hysterophorus* and the native vegetation of *C. dactylon* [43,63]. Co-occurrence network analysis revealed that in bacterial networks, the *C. dactylon* treatment exhibited the highest complexity (dominated by *Chloroflexi* and *Acidobacteriota*), while in fungal networks, both *P. hysterophorus* and C. dactylon treatments showed higher numbers of nodes and connections (primarily represented by *Ascomycota* and *unclassified fungi*) [24,32]. This suggests that both invasive plant of *P. hysterophorus* and native plant of *C. dactylon* may enhance the complexity of ecological networks by promoting microbial interactions, while simultaneously potentially reducing community stability [19,26,41]. In summary, vegetation type is a key factor regulating soil microbial communities, likely through modifying the availability of environmental resources and altering microbial interaction patterns, thereby shaping the structure and function of soil microbial assemblages [27,40,46].

### 4.3. Influence Factors of CCA, Mantel Test and Intermediary Analysis

In the WFLZ ecosystem, soil microbial communities are influenced by plant biomass diversity and anthropogenic farming practices [38,50]. The CCA in this study revealed that soil physicochemical properties and enzyme activities collectively explained less than 30% of the variance in fungal and bacterial communities (Figure 10), suggesting that unmeasured factors may also significantly affect microbial community composition and assembly [31,42]. Previous research had indicated that the composition of microbial communities is closely linked to plant communities and soil nutrient cycling [6,10]. As key soil enzymes, urease and alkaline phosphatase (ALP) are directly involved in the mineralization of organic nitrogen and phosphorus, respectively [21]. Their activity levels reflect the turnover rate of soil nutrient availability, thereby selecting for bacterial taxa capable of rapidly responding to and utilizing these readily available nutrients [11,25]. In this study, urease, available phosphorus, BD, and ALP were significantly correlated with bacterial community assembly. This aligns with earlier findings that soil enzyme activities (such as urease and ALP) directly shape microbial functional groups by regulating nitrogen and phosphorus cycling [56,63]. In contrast, bacterial and fungal communities showed a weaker correlation with urease but a stronger association with AN. This may indicate that fungi are more directly influenced by the total soil inorganic nitrogen pool (AN) rather than the instantaneous rate of nitrogen transformation [13,60]. At the same time, BD again emerged as a core driver of fungal community structure, suggesting that suitable soil bulk density not only affects soil aeration but also provides a physical substrate for fungal hyphal extension and colonization [14]. Notably, SMC, a key factor in WFLZ, had a stronger effect on bacterial communities than on fungal communities, implying that the flooding–drying cycles characteristic of WFLZ significantly influence bacterial composition [3,7]. Furthermore, the influence of AP and SOC on fungal communities should not be overlooked, indicating that in the WFLZ area, fungal community composition is more dependent on soil nutrient status [23,32].

In the WFLZ ecosystem, the drivers of microbial community stability and their ecological strategies vary significantly across vegetation types [19,27]. Mantel test analysis revealed that bacterial stability in both *C. dactylon* and *P. hysterophorus* communities was significantly influenced by SMC, while fungal diversity in *P. hysterophorus* was also affected by TK and CAT [53,61]. In contrast, the stability of bacterial and fungal communities in the *Z. mauritiana* and *Z. Mays* treatments was more strongly shaped by soil enzyme activities than by SMC. This divergence likely stems from differences in microbial ecological strategies among plant communities [45,67]. Furthermore, in *P. hysterophorus* and *C. dactylon*, located in the WFLZ, microbial stability was predominantly regulated by SMC. In these two communities, bacteria occupied the dominant ecological niche and exhibited higher co-occurrence network complexity (Figure 9). Increased bacterial richness intensified interspecific competition, thereby reducing bacterial community stability, yet indirectly enhancing fungal stability [25]. Consequently, water-level fluctuations had a relatively minor impact on fungal stability in *P. hysterophorus* and *C. dactylon* [54,65].

The mediation model further indicated that in the *P. hysterophorus* community, SMC indirectly affected bacterial stability by altering TK and pH, whereas in *C. dactylon*, SMC influenced bacterial stability primarily through pH modulation [27,41]. On the other hand, in the *Z. mays* community, bacterial stability was less affected by water-level fluctuations and relied more heavily on soil enzyme activities [21,60]. This was corroborated by the mediation model, which showed that both bacterial and fungal stability in *Z. mays* were positively influenced by enzyme activities but negatively affected by SMC [51,67]. Meanwhile, in the *Z. mauritiana* community, microbial stability was not influenced by SMC but was directly regulated by pH; available potassium also directly affected fungal stability through synergistic interactions with pH and CAT [23,47]. These findings highlight clear differences in the relative importance of soil moisture versus enzyme activities in regulating microbial community stability across vegetation types in the drawdown zone [13,25]. Riparian communities were more susceptible to moisture fluctuations, while upland and non-drawdown zone communities depended more strongly on enzyme-driven nutrient cycling processes [11,21,55]. Future studies integrating metagenomic and transcriptomic approaches could provide deeper insights into the metabolic responses of niche-specific microorganisms under changing water levels, as well as differences in microbial interactions between invasive and native plants, thereby offering a theoretical basis for ecological restoration and vegetation management in the WFLZ [7,27,63].

## 5. Conclusions

In this study, we choose four typical plant communities (*P. hysterophorus*, *Z. mauritiana*, *C. dactylon*, *Z. mays*) in a water-level-fluctuation zone of the Lower Jinsha River, which explored the changing characteristics of soil nutrient and microbial community, especially bacteria and fungi, and their driving role in the microbial stability in four typical plant communities. Overall, this result indicated that soil properties and enzymes noticeably changed among the four types of different plant communities in the WLFZ, of which the *P. hysterophorus* and *Z. mauritiana* treatments were eventually higher than those of the *Z. mays* and *C. dactylon* treatments. In the bacteria and fungi community, the OTU number of *P. hysterophorus* and *Z. mauritiana* treatments was higher than that of the *C. dactylon* and *Z. mays* treatments, for which the LEfSe showed that the bacterial biomarkers only explained the order, but the fungal biomarkers could explain species. The bacterial and fungal diversity among the four types of different plant communities in the WLFZ significantly changed such that the bacterial and fungal explained for the PCoA was at 42.45% and 28.17%, respectively, and the anosim analysis of bacteria and fungi showed the *p* was 0.001 and the R was at 0.6995 and 0.7491. The bacterial and fungal co-occurrence network patterns presented that the bacterial community structure of the *C. dactylon* and *P. hysterophorus* treatments were more complicated than those of the *Z. mauritiana* and *Z. mays* treatments, whereas the community stability of *C. dactylon* and *P. hysterophorus* treatments were notably lower than those of the *Z. mauritiana* and *Z. mays* treatments. The result of CCA indicated that the urease, AP, BD and ALP held a significant correlational relationship with bacterial community of the four different communities, and the BD, AN and ALP had a marked correlation with fungi of different communities (*p* < 0.05). Correlation analysis demonstrated that SMC and pH serve as key factors significantly influencing microbial community stability and diversity across all four vegetation types, while other soil properties exhibit community-specific effects. The mediation model revealed distinct and community-specific regulatory pathways of bacterial and fungal stability, showing that pH served as the primary direct driver in the *Z. mauritiana* community, soil moisture exerted dominant effects in *Z. mays* and *P. hysterophorus*, while in *C. dactylon*, bacterial stability was indirectly modulated by pH mediated through SMC changes. In conclusion, the WLFZ is a fragile yet crucial ecosystem that demands careful management to balance human needs with ecological health.

## Figures and Tables

**Table 1:** Overview of the experimental site in the WLFZ.

Site Name	Dominant Plant	Longitude and Latitude	Elevation/m	Slope/°	Community Overview
*Z. mays*	*Zea Mays* L.	101.867725° E, 25.897274° N	964.91	2	*Zea mays* cover is about 90–95% and also includes weeds such as *Echinochloa colona*, *Paspalidium flavidum*, and *Boerhavia diffusa*.
*Z. mauritiana*	*Ziziphus mauritiana* Lam	101.870133° E, 25.900264° N	965.36	2	*Ziziphus mauritiana* cover is about 85–90% and also includes weeds such as *Digitaria Halle*, *Paspalidium flavidum*, *Boerhavia diffusa*
*P. hysterophorus*	*Parthenium hysterophorus* L.	101.86866° E, 25.897404° N	963.28	3	*Parthenium hysterophorus* cover is about 85–90%, and height is about 80–120 cm and includes weeds such as *Cynodon dactylon*, Malvastrum coromandelianum, Digitaria Haller.
*C. dactylon*	*Cynodon dactylon* (Linn.) Pers.	101.870025° E, 25.897339° N	963.16	1	*Cynodon dactylon* cover is about 95–97%, and height is about 5~7 cm and includes weeds such as Paspalidium flavidum.

**Table 2:** Prime names and primer sequence of target gene.

Microbiological Type	Amplification Region	Primer Name	Primer Sequences
bacteria	V3V4	338F	ACTCCTACGGGAGGCAGCAG
		806R	GGACTACHVGGGTWTCTAAT
fungi	ITS	ITS1F	CTTGGTCATTTAGAGGAAGTAA
		ITS2R	GCTGCGTTCTTCATCGATGC

**Table 3:** α diversity index of soil bacteria and fungi under different communities of four types (Mean ± SE).

Sites	Richness	Shannon– Wiener	Simpson	Margalef	Pielou	Chao 1
*C. D.* bacteria	3408.33 ± 125.88 b	6.78 ± 0.07 ab	0.996 ± 0 ab	319.30 ± 11.79 b	0.83 ± 0.01 ab	3873.8 ± 118.5 b
*Zi. M.* bacteria	3096.17 ± 52.14 c	6.64 ± 0.02 b	0.996 ± 0 ab	291.40 ± 5.20 bc	0.83 ± 0.00 bc	3599.73 ± 69.09 bc
*P. H.* bacteria	3811.83 ± 87.58 a	6.95 ± 0.05 a	0.997 ± 0 a	359.31 ± 8.50 a	0.84 ± 0.01 a	4313.08 ± 89.1 a
*Ze. M.* bacteria	2862.17 ± 129.00 c	6.45 ± 0.09 c	0.995 ± 0 b	268.35 ± 11.96 c	0.81 ± 0.01 c	3395.23 ± 117.48 c
*C. D.* fungi	480.33 ± 40.91 b	3.87 ± 0.17 a	0.94 ± 0.01 a	304.40 ± 10.19 b	0.48 ± 0.02 a	503.04 ± 46.56 b
*Zi. M.* fungi	351.33 ± 13.38 c	3.78 ± 0.26 a	0.93 ± 0.03 a	281.47 ± 4.27 bc	0.47 ± 0.03 a	363.31 ± 11.58 c
*P. H.* fungi	627.83 ± 31.93 a	3.66 ± 0.10 a	0.91 ± 0.02 a	335.03 ± 8.07 a	0.44 ± 0.01 a	686.14 ± 38.407 a
*Ze. M.* fungi	406.67 ± 42.88 bc	3.75 ± 0.26 a	0.92 ± 0.02 a	258.82 ± 11.61 c	0.47 ± 0.03 a	418.46 ± 43.81 bc

Note: Letter differences indicate significant differences, i.e., *p* < 0.05.

## Data Availability

The original contributions presented in this study are included in the article. Further inquiries can be directed to the corresponding authors.
